# Stunted Growth Caused by Blast Disease in Rice Seedlings Is Associated with Changes in Phytohormone Signaling Pathways

**DOI:** 10.3389/fpls.2017.01558

**Published:** 2017-09-06

**Authors:** Chang-Jie Jiang, Xiao-Long Liu, Xin-Qiong Liu, Hui Zhang, Ying-Jie Yu, Zheng-Wei Liang

**Affiliations:** ^1^Institute of Agrobiological Sciences, National Agriculture and Food Research Organization Tsukuba, Japan; ^2^Northeast Institute of Geography and Agroecology, Chinese Academy of Sciences Changchun, China; ^3^College of Life Science, South-Central University for Nationalities Wuhan, China

**Keywords:** phytohormones, growth, defense, trade-off, *Magnaporthe oryzae*, rice

## Abstract

In response to pathogen attack, plants prioritize defense reactions generally at the expense of plant growth. In this work, we report that changes in phytohormone signaling pathways are associated with the stunted plant growth caused by blast disease in rice seedlings. Infection of rice seedlings with blast fungus *Magnaporthe oryzae* (race 007.0) at the four-leaf stage (three true leaves) resulted in considerable inhibition of the growth of the upper uninfected distal leaves; the length of leaf blade and leaf sheath of the sixth and seventh leaf was reduced by 27 and 82%, and 88 and 72%, respectively, compared to that in the uninoculated plant control. Interestingly, cutting off the blast-infected fourth leaf blade within 2 days post inoculation (dpi) significantly rescued the inhibition of leaf growth, implying that an inhibitory substance(s) and/or signal was generated in the blast-infected leaves (fourth leaf) and transmitted to the upper distal leaves (sixth and seventh) during the 2-dpi period that induced growth inhibition. Expression analysis of marker genes for phytohormone pathways revealed acute activation of the jasmonate (JA) and abscisic acid (ABA) signaling pathways, and repression of auxin, gibberellic acid (GA) and salicylic acid (SA) signaling pathways, in the sixth leaf. The genes related to cell wall expansion were also significantly downregulated. In the blast-infected fourth leaf, JA pathway was activated within 2 dpi, followed by activation of ABA pathway 3 dpi. Further, leaf inhibition caused by blast infection was partially rescued in the rice mutant line *coleoptile photomorphogenesis 2* (*cpm2*), which is defective in the gene encoding allene oxide cyclase (*OsAOC*). These results indicate that the JA signaling pathway is at least partly involved in the growth inhibition processes. Collectively, our data suggest that, upon pathogen attack, rice seedlings prioritize defense reactions against the infecting pathogen by temporarily ceasing plant growth through the systemic control of phytohormone pathways.

## Introduction

Plants have evolved a set of mechanisms to tune the balance of plant growth and defense reactions for better survival and fitness in nature. In response to pathogen attack, plants allocate more resources toward defense reactions, while temporarily limiting the supply of nutrients and energy resources to less urgent physiological processes such as photosynthesis and plant growth (Huot et al., 2014; Takatsuji, 2017). Consequently, defense responses negatively impact plant fitness because of their usage of highly costly resources. Therefore, plants generally restore the balance to favor growth and development in the absence of pathogen challenges (Huot et al., 2014; Takatsuji, 2017). In addition, rather than deploying defense mechanisms uniformly across different tissues, plants prioritize protecting the tissues that contribute more to a plant’s future fitness, such as young sink tissues and reproductive structures (optimal defense hypothesis, ODH) (Meldau et al., 2012; Meldau and Baldwin, 2013). While the molecular mechanisms remain largely unknown, recent studies have implicated that a complex signaling network involving phytohormones plays a major role in such a trade-off between growth and defense (Huot et al., 2014; Kazan and Lyons, 2014; Takatsuji, 2017). Phytohormones play central signaling roles in diverse biological processes including plant growth and development, as well as defense responses. Each of the hormones generates and transmits a distinct growth and/or defense signal, while crosstalk between them has been shown to be essential for the outcome of plant–pathogen interactions (Robert-Seilaniantz et al., 2011; Yang et al., 2013; Huot et al., 2014; Takatsuji and Jiang, 2014). It has been well documented that salicylic acid (SA), jasmonates (JA), and ethylene (ET) play key roles in activation of defense responses to various pathogens (Robert-Seilaniantz et al., 2011; Yang et al., 2013; Huot et al., 2014; Takatsuji and Jiang, 2014). It was shown that a heat shock factor-like transcription factor, TBF1, plays a key role in the growth-to-defense transition in response to SA and the MAMP signal, elf18, in *Arabidopsis* (Pajerowska-Mukhtar et al., 2012). Other growth-regulating hormones, such as auxin, gibberellic acid (GA), cytokinins (CKs), and abscisic acid (ABA), also have an important part to play in plant–pathogen interactions via cooperative or antagonistic crosstalk with the defense hormones, SA, JA, and ET (Robert-Seilaniantz et al., 2011; Yang et al., 2013; Takatsuji and Jiang, 2014; Ma and Ma, 2016).

In rice, SA has been implicated in activation of defense responses to various pathogens including blast fungus *Magnaporthe oryzae* and leaf–blight bacteria *Xanthomonas oryzae* pv. *oryzae* (Schweizer et al., 1999; Rohilla et al., 2002; Babu et al., 2003; Yang et al., 2004; Shimono et al., 2007; Sugano et al., 2010). The SA signaling in rice is mediated by two downstream factors, OsNPR1 and WRKY45 (Shimono et al., 2007; Sugano et al., 2010), unlike that in *Arabidopsis* where it is primarily mediated by NPR1 (Wang et al., 2006). JA and ET are also involved in resistance to rice pathogens *M. oryzae*, *X. oryzae* pv. *oryzae*, and *Rhizoctonia solani* (Iwai et al., 2006; Mei et al., 2006; Bailey et al., 2009; Seo et al., 2011; Yamada et al., 2012; Helliwell et al., 2013; Riemann et al., 2013). In contrast to the mostly antagonistic interaction between SA- and JA-mediated signaling pathways in *Arabidopsis*, it was shown that SA and JA activate a common defense system in rice (Brooks et al., 2005; Laurie-Berry et al., 2006; Robert-Seilaniantz et al., 2011; Garg et al., 2012; Tamaoki et al., 2013). The growth hormones auxin and GAs have been shown to negatively affect rice resistance to *M. oryzae*, *X. oryzae* pv. *Oryzae*, and *Xanthomonas oryzae* pv. *oryzicola* (Tanaka et al., 2006; Ding et al., 2008; Yang et al., 2008; Domingo et al., 2009; Fu et al., 2011). Auxin was shown to upregulate expansin gene expression, leading to cell wall loosening, and thus rendering the plant more susceptible to pathogen invasions (Ding et al., 2008; Domingo et al., 2009). It was further shown that overexpression of *OsNPR1* in rice plants resulted in growth attenuation (dwarf phenotype) by repressing auxin signaling pathway through upregulating *OsGH3.8*, a gene encoding IAA-amino synthase (Li et al., 2016). Yang et al. (2012) reported that JA antagonistically interacts with GA signaling cascade to prioritize defense over growth upon pathogen attacks in both *Arabidopsis* and rice. We previously showed that CKs and ABA interact with SA cooperatively and antagonistically, respectively, in rice–*M. oryzae* interaction (Jiang et al., 2010, 2013).

It has been shown that rice plants diseased by virus and blast fungus exhibit growth stunting. Rice infection by *rice stripe virus* (RSV) (Satoh et al., 2010), *rice dwarf virus* (RDV) (Satoh et al., 2011), *rice grassy stunt virus* (RGSV) (Satoh et al., 2013) and *rice tungro spherical virus* (RTSV) (Budot et al., 2014) result in a severe growth stunting. Molecular analysis revealed that the virus infection-induced growth inhibition is associated with suppression of GA and/or auxin signaling cascades and cell wall synthesis and expansion (Satoh et al., 2010, 2011, 2013; Budot et al., 2014). On the other hand, several decades ago, it was observed that infection of rice seedlings with blast fungus *M. oryzae* results in a severe growth inhibition in addition to formation of blast disease lesions at the infection sites; this morphological symptom is called “Zurikomi” that means stunting of plant growth in Japanese (Tokunaga et al., 1959). The growth inhibition is manifested the most in successive upper two to three leaves counted from the infected leaf, especially in the early stages of plant growth (Tokunaga et al., 1959; Yoshida et al., 1992). Microscopic observation suggested that the suppression of cell division is a major factor for stunting of blast-infected plants (Yoshida et al., 1992). It was initially thought that the growth stunting is caused by excess accumulation of coumarin within plants triggered by the blast fungal toxin piricularin (Tamari and Kaji, 1959a,b). This, however, could not be confirmed in later studies (Satoh and Kozaka, 1966, 1971). Moreover, ethylene evolution was detected from blast inoculated rice plants (Kozaka and Teraoka, 1977), but which was found not be associated with growth stunting of blast-infected rice plants (Yoshioka et al., 1992). Thus, the detailed mechanism of the growth stunting in blast-infected rice plants remains largely unknown.

In this study, we report that a yet unknown inhibitory substance(s) and/or signal(s) is generated in the *M. oryzae*-infected leaves and transmitted to the upper distal leaves, where it activates JA and ABA, while suppressing GA and auxin signaling pathways, and consequently causing an inhibition of growth in the leaves. These findings provide new insights into the controlling mechanism of growth-defense balance in plants.

## Materials and Methods

### Plant Materials and Measurements

The Japonica rice cultivar ‘Nipponbare’ was used in this study. Seeds were germinated in soil (Bonsol No. 2; Sumitomo Chemical Corp., Tokyo, Japan) in plastic pots (50 mm square × 50 mm deep, and a drainage hole), four seeds per pot, and the seedlings were grown in a greenhouse at 28°C in the day (14 h) and 23°C in the night (10 h). The relative humidity in the greenhouse was approximately 70%.

A rice mutant line *coleoptile photomorphogenesis 2* (*cpm2*) defective in the gene encoding allene oxide cyclase (*OsAOC*) (Biswas et al., 2003) and its wild-type (WT) rice line ‘Nihonmasari’ were used for investigation of the role of JA in growth stunting of blast-infected rice plants. The homozygous mutant seedlings were selected from a heterozygous population based on phenotype of elongated shoots compared with WT. The homozygosity of mutant seedlings was further confirmed by PCR genotyping, using primer set, 5′-ACGAACATCTCCTGCACCTT-3′ and 5′-CTCGCGAGTCTCCGTCAG-3′.

About 4–5 weeks after blast inoculation, the fully grown fifth to seventh leaves were detached from shoot bases, and measured for lengths of leaf blades and leaf sheaths with a scaled ruler.

### Pathogen Culture and Inoculations

Culture and inoculations of the blast fungus *M. oryzae* (race 007.0) were carried out according to Akagi et al. (2015). Briefly, the fungus was grown on an oatmeal agar medium (30 g/L oatmeal, 5 g/L sucrose, and 16 g/L agar) at 26°C for 10–12 days. Conidia formation was induced by irradiation under continuous black–blue light (FL15BLB; Toshiba, Osaka, Japan) at 24°C for 2–4 days. The conidia were suspended in 0.02% Silwet L-77 at a density of 1–2 × 10^5^/mL and sprayed onto rice plants at the four-leaf stage. As a mock treatment control, the same volume of 0.02% Silwet L-77 was sprayed. After incubation in a dew chamber at 24°C for 24 h, the rice plants were moved back to the greenhouse.

### Gene Expression Analysis

Rice seedlings at the four-leaf stage were blast-inoculated, and the inoculated fourth leaf blades of half the plants was cut off at 2 dpi. Sixth whole leaf was collected at 3 dpi, and leaf blades and leaf sheathes separately at 6 dpi. Three biological replicates were collected, four leaves in each replicate.

Real time-polymerase chain reaction (RT-PCR) was used to analyze the samples for expression of marker genes for JA (JAmyb and OMT), ABA (SalT and OsWsi18), auxin (ARF1 and IAA9), GA (OsGA2ox3 and OsGA20ox1) and SA (WRKY45 and OsNPR1), and PR genes OsPR1b and PBZ1. The genes and primer sequences used for qRT-PCR are listed in Supplementary Table 1.

Total RNA was isolated using the TRIzol reagent (Invitrogen) and reverse-transcribed by using ReverTra Ace (TOYOBO, Osaka, Japan) according to the manufacturer’s protocol. Quantitative RT-PCR (qRT-PCR) was run on a Thermal Cycler Dice TP800 system (Takara Bio) using SYBR premix ExTaq mixture (Takara Bio) as previously described (Shimono et al., 2007).

### Phytohormone Treatments and Measurements

All stock solutions, except brassinolide (BR), were prepared at a concentration of 100 mM as described previously (Jiang et al., 2009). BR was prepared at 20 mM concentration. Indole-3-acetic acid (IAA; Sigma, St. Louis, MO, United States), gibberellin A3 (GA3; Wako, Osaka, Japan), ABA [(±)-*cis*–*trans*, Sigma], methyl jasmonate (ME-JA; Wako), and brassinolide (BR; Wako) were dissolved in absolute ethanol. Kinetin (Sigma) and benzothiadiazole *S*-methyl ester (BTH; Wako) were dissolved in dimethyl sulfoxide (DMSO); and 1-aminocyclopropane-1-carboxylic acid (ACC; Sigma) and sodium salicylate (SA; Nacalai Tesque, Tokyo, Japan) in H_2_O.

For plant treatments, rice seedlings at four-leaf stage (three true leaves) were transferred to a container containing each of the phytohormone solutions at 50 μM for IAA, GA3, CK, ACC, ABA, JA, and BTH, and at 10 μM for BR. Water was used as mock control. The rice seedlings were further grown for 4 weeks, and leaf lengths of the fifth leaf were measured.

Measurement of JA and ABA content were performed as described previously (Kojima and Sakakibara, 2012).

### Experimental Design and Data Analysis

All the experiments were conducted with three replicates each consisting of one to two planting pots with four plants per pot for each inoculation. The plants for each replicate were placed in a separate container, and the three replicate containers were rotated within the growth chamber every 2–3 days to minimize any effect of location. All the experiments were repeated at least twice independently; these produced similar results, so data from only one trial are presented.

Statistical analyses were performed using the statistical software SPSS 21.0 (IBM Corp., Armonk, NY, United States). One-way analysis of variance (ANOVA) was used to compare differences in the means among treatments (*P* = 0.05).

Calculations of mean values and standard deviations, graph plotting and correlation analysis were performed using Microsoft Office 365-Excel software (Microsoft Corporation, Tokyo, Japan).

## Results

### Blast Infection Caused Growth Inhibition of Rice Seedlings

**FIGURE 1:**
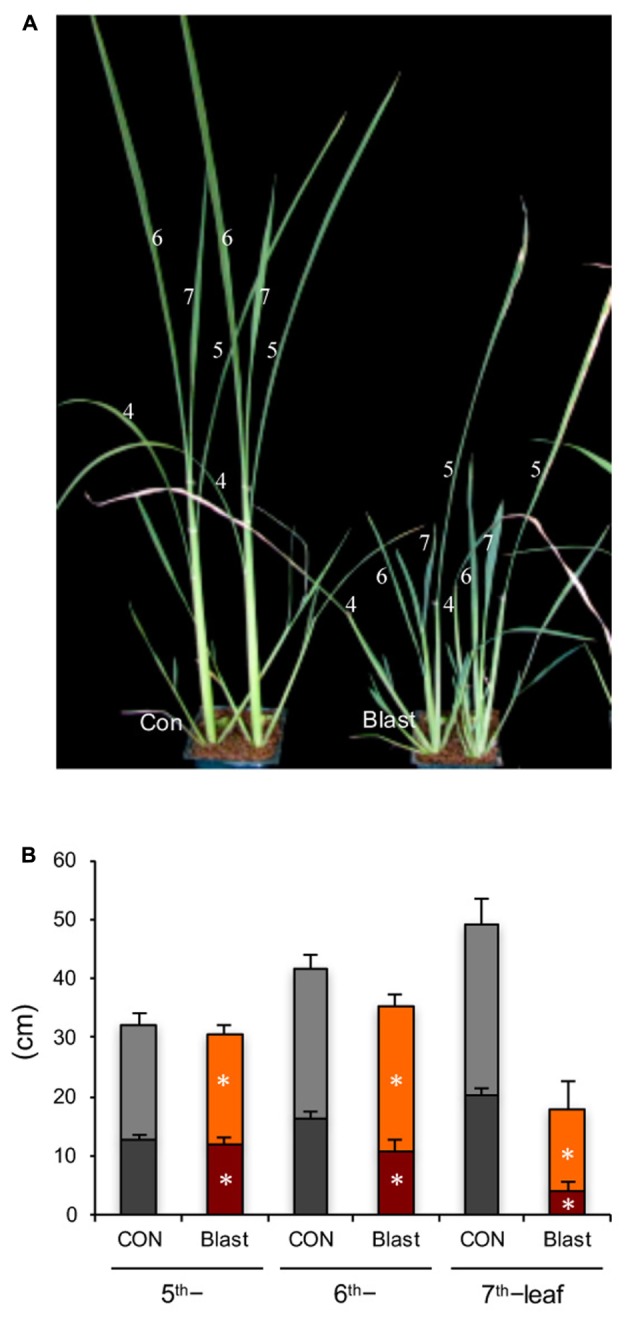
Blast infection causes stunted growth of rice seedlings. **(A)** Images of mock control (left) and blast-infected (right) rice seedlings. Rice seedlings at the four-leaf stage were inoculated with blast fungus *M. oryzae* (race 007.0), and the photograph was taken at 12-dpi. Numbers indicate leaf positions counted from shoot base. **(B)** Lengths of leaf blades (upper column) and leaf sheathes (bottom column) of fifth to seventh leaves, measured at 5 weeks post inoculation (5 wpi). Values are the means ± standard errors; the asterisks indicate significant difference from the mock control plants (*t-*test, ^∗^ indicates *P* < 0.01).

**FIGURE 2:**
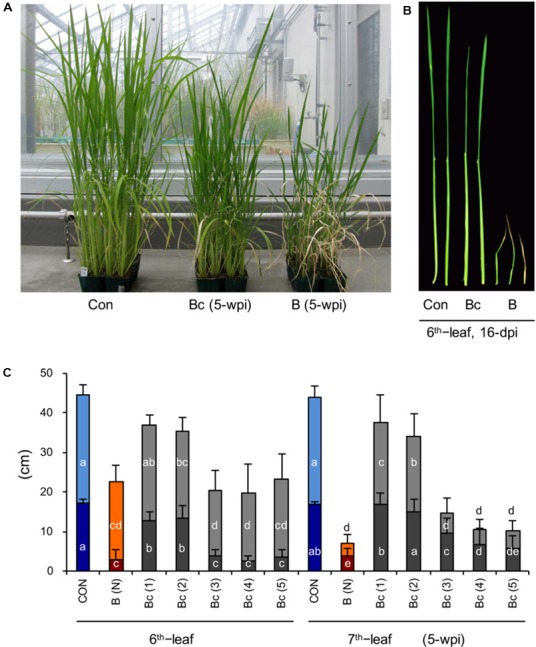
Cutting off the blast-infected leaf blades significantly rescues the stunted growth of upper distal leaves. **(A)** Image of rice seedlings of mock control (Con, left), blast-infected (**B**, right), and cutting off blast-infected leaf blades (Bc, middle). Rice seedlings at the four-leaf stage were inoculated with blast fungus *M. oryzae* (race 007.0), and the photograph was taken at 5 wpi. **(B)** Close observation of the sixth leaf in mock control (Con, left), blast-infected (**B**, right), and cutting off blast-infected leaf blades (Bc, middle) at 16-dpi. **(C)** Lengths of leaf blades (upper column) and leaf sheathes (bottom column) of sixth and seventh leaves of rice seedlings shown in **(A)**. Blast-infected leaf blades were cut off at dpi as indicated by numbers in parentheses. (N) depict no cutoff of leaf blades. Values are the means ± standard errors; different letters on the bars indicate a significant difference (*P* < 0.05) based on Duncan’s test.

Rice seedlings at four-leaf stage, with fourth leaves fully expanded, were inoculated with a compatible blast fungus race 007. Visible blast disease lesions appeared on the fourth leaf blades at 3 dpi and the fourth leaf blades were wilted by 7 dpi (data not shown). From then on, upper leaves above the inoculated fourth leaves (N) exhibited severe growth stunting; this was particularly true for both leaf blades and leaf sheathes, in N+2–3 leaves (sixth and seventh leaves) (**Figures 1A,B**). As shown in **Figure 2B**, sixth leaf in the infected plants was greatly suppressed, and the distal-half of most leaf blades was dead and dried. These results are consistent with previous observations by Tokunaga et al. (1959) and Yoshida et al. (1992). The growth inhibition was slight and negligible in N+4 and upper leaves, consistent with observation by Yoshida et al. (1992).

### Cutting off Blast-Infected Leaf Blades Significantly Rescued the Growth Inhibition

We hypothesized that a growth inhibition substance and/or signal is generated in the blast-infected leaves and transmitted to upper distal leaves. To determine this, we cut off the inoculated fourth leaf blades with a scissor in times at 1, 2, 3, 4, 5, 6, and 7 dpi, and observed the effect on plant growth. The results show that cutting off the blast-infected leaf blades within 48 h after inoculation can significantly rescue the growth of plants (**Figures 2A–C**), particularly N+2–3 leaves (sixth and seventh leaves) (**Figures 2B,C**). Cutting off blast-infected leaves after 48 h had little rescue effect on the leaf growth (**Figure 2C**).

### Jasmonate Signaling Is Activated in Blast-Infected Leaves

**FIGURE 3:**
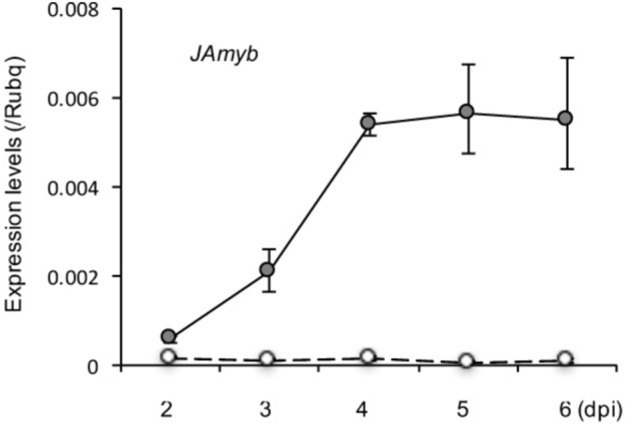
Transcriptional induction of JA marker gene, *JAmyb*, in response to blast infection in the blast-infected leaf blades. Rice seedlings at the four-leaf stage were inoculated with blast fungus *M. oryzae* (race 007.0). Transcript levels of JA-responsive genes, *JAmyb*, were determined at the indicated time points (dpi) by qRT-PCR using *Rubq1* as an internal control. Open and filled circles depict mock control and blast-inoculation, respectively. Mean values of three biological replicates with standard deviations are shown.

We previously reported activations of SA, ABA, and CK signaling pathways in blast-infected leaf blades of rice seedlings (Jiang et al., 2010, 2013). To determine the changes in JA signaling pathway during blast infection, we analyzed expression of JA-responsive marker gene *JAmyb* (Lee et al., 2001) in blast-inoculated fourth leaf blades in a time course manner up to 6 dpi. We found that *JAmyb* is significantly upregulated by blast infection within 2 dpi, and peaked at 4 dpi and retained the upregulation until the end of the time course (**Figure 3**).

### Distal Changes in Phytohormone Signaling Pathways

**FIGURE 4:**
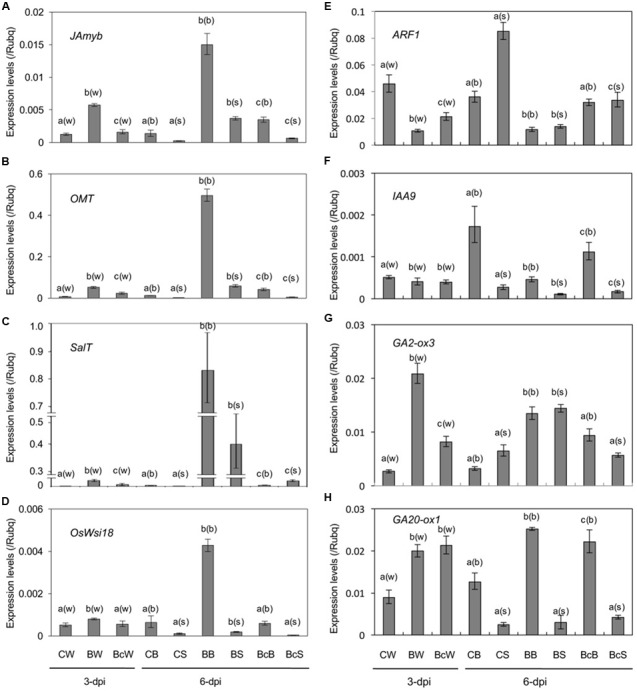
Expression analysis of marker genes for JA **(A,B)**, ABA **(C,D)**, IAA **(E,F)**, and GA **(G,H)** in the sixth leaf of mock control (CW, CB, and CS), blast-infected (BW, BB, and BS), and cutting off blast-infected leaf blades (BcW, BcB, and BcS). Rice seedlings at four-leaf stage were blast-inoculated, and half of them were subjected to cutting off the inoculated fourth leaf blades at 2-dpi. Whole sixth leaf from every treatment was collected at 3-dpi (CW, BW, and BcW), and leaf blades (CB, BB, and BcB) and leaf sheathes (CS, BS, and BcS) separately at 6-dpi. Values are the means ± standard errors. Different letters above bars indicate a significant difference (*P* < 0.05) based on Duncan’s test; (w): for whole leaves, (b): for leaf blades, (s): for leaf sheaths.

To obtain insight into the growth stunting by blast infection, we examined changes in phytohormone pathways in the sixth leaf (N+2) by expression analysis of hormone-responsive marker genes (**Figure 4** and Supplementary Table 1). The results indicated activation of stress hormone pathways, and suppression of growth promoting hormone pathways in blast-infected plants (**Figure 4**).

Jasmonate-responsive genes, *JAmyb* (Lee et al., 2001) and *OMT* (Yoshii et al., 2010), and ABA-responsive genes, *SalT* (Rabbani et al., 2003) and *OsWsi18* (Joshee et al., 1998), were significantly upregulated in response to blast infection at 3 dpi, and the expression levels were further increased at least until 6 dpi (**Figures 4A–D**). Interestingly, cutting off the inoculated fourth leaf blades reduced the induction of these genes (**Figures 4A–D**). Determination of JA and ABA contents in the sixth whole leaf at 3 dpi showed no significant differences between uninoculated control and blast-infected plants (Supplementary Table 2).

By contrast, auxin-responsive genes, *ARF1* (Waller et al., 2002) and *IAA9* (Jain et al., 2006), were downregulated in response to blast infection, and cutting off the inoculated leaves partially restored the downregulation (**Figures 4E,F**). On the other hand, a significant upregulation of GA-inactivation gene *OsGA2ox3* (Sakai et al., 2003) and GA biosynthesis gene *OsGA20ox1* (Toyomasu et al., 1997) were observed in blast-infected plants (**Figures 4G,H**). Cutting off the inoculated leaves partly reduced the upregulation (**Figures 4G,H**). *OsGA2ox3* encodes an active GA 2-oxidase that inactivates bioactive GAs and its immediate precursors (Sakai et al., 2003); and *OsGA20ox1* encodes a key enzyme for GA biosynthesis, whose expression is negatively regulated by GA (Toyomasu et al., 1997; Sakamoto et al., 2001). These results indicate that signaling pathways of auxin and GA in the upper distal leaves are downregulated by blast infection.

**FIGURE 5:**
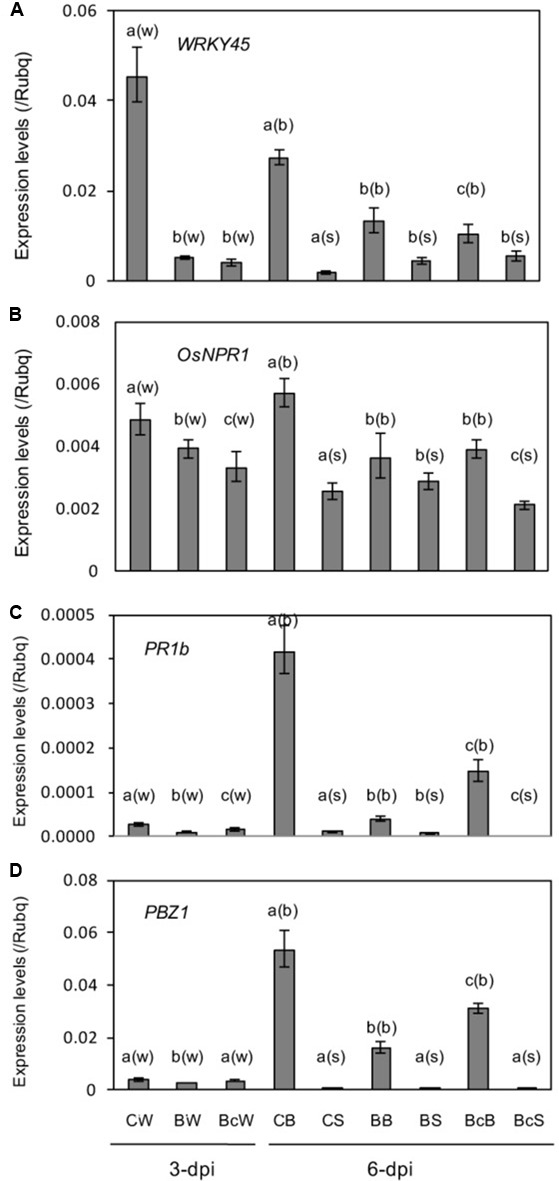
**(A–D)** Expression analysis of SA responsive genes and PR genes in the sixth leaf as described in **Figure 4**. Values are the means ± standard errors. Different letters above bars indicate a significant difference (*P* < 0.05) based on Duncan’s test; (w): for whole leaves, (b): for leaf blades, (s): for leaf sheaths.

Contrary to expectation, SA responsive marker genes *WRKY45* (Shimono et al., 2007) and *OsNPR1* (Sugano et al., 2010), and PR genes *OsPR1b* and *PBZ1*, were downregulated in response to blast infection (**Figures 5A,B**). Cutting off the infected leaf blades slightly restored expression of *OsPR1b* and *PBZ1* in leaf blades, while no appreciable effect on *WRKY45* and *OsNPR1* was observed (**Figures 5C,D**). Expression levels of the genes were relatively low and had no appreciable restoration by cutting off the infected leaf blades (**Figures 5C,D**).

### Cell Wall Related Genes Are Downregulated

**FIGURE 6:**
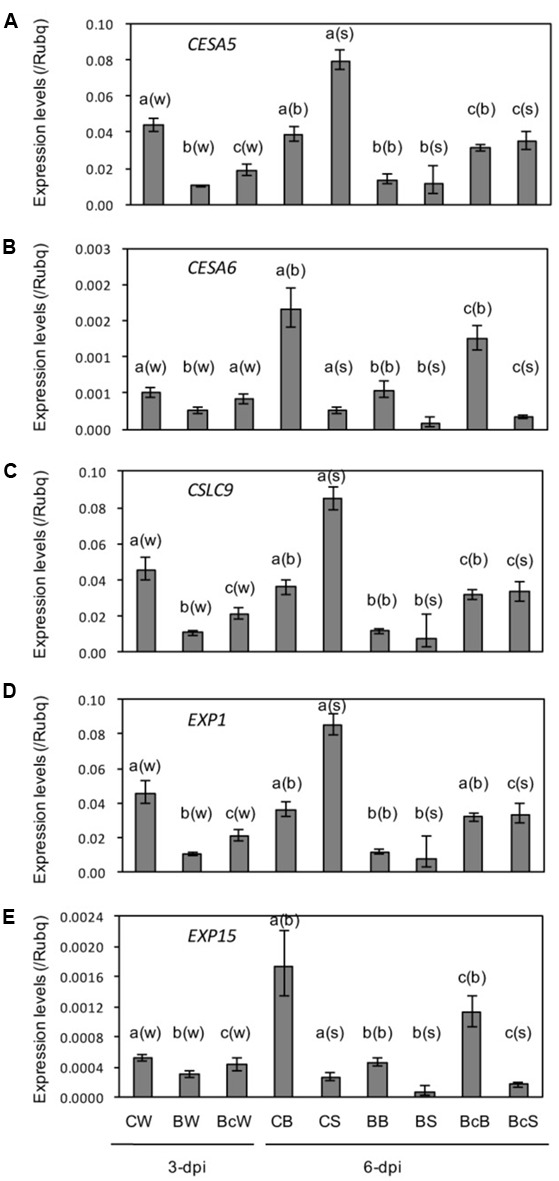
**(A–E)** Expression analysis of cell wall-related genes in sixth leaf as described in **Figure 4**. Values are the means ± standard errors. Different letters above bars indicate a significant difference (*P* < 0.05) based on Duncan’s test; (w): for whole leaves, (b): for leaf blades, (s): for leaf sheaths.

Cellulose synthase genes *OsCESA5* and *OsCESA6*, cellulose synthase-like gene *OsCSLA9*, and expansin *OsEXP1* and *OsEXP15* were downregulated in response to blast infection, and cutting off the inoculated leaves partially reduced the downregulation (**Figures 6A–E**). These results are similar to those in stunted rice plants infected with RTSV (Budot et al., 2014).

### Reduction in JA Levels Partially Rescued the Growth Stunting

**FIGURE 7:**
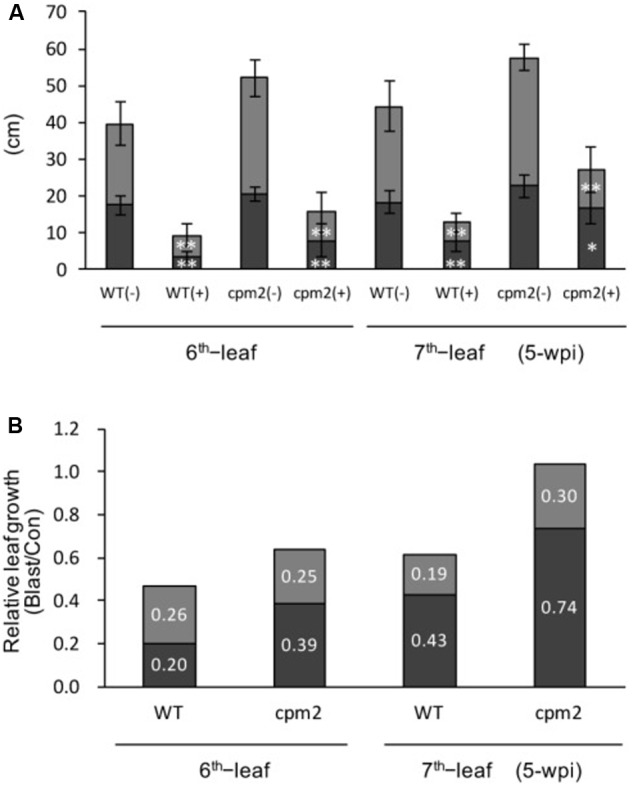
Growth inhibition by blast infection is partially rescued in JA-deficient mutant *cpm2*. **(A)** Rice seedlings at the four-leaf stage were inoculated with blast fungus *M. oryzae* (race 007.0), and lengths of leaf blades (upper column) and leaf sheathes (bottom column) of sixth and seventh leaves were measured at 5-wpi. WT: wild type control of *cpm2*; (–) = mock control; (+) = blast-infected. Values are the means ± standard errors; the asterisks indicate significant difference from the mock control plants (*t-*test, ^∗^ and ^∗∗^ indicate *P* < 0.05 and 0.01, respectively). **(B)** Relative growth of leaf blades (upper columns) and leaf sheaths (bottom columns) of sixth and seventh leaves were expressed as leaf length in blast-infected plants relative to that in mock control plants.

As JA responsive marker genes were significantly upregulated in the stunted leaves (**Figures 4A,B**), a rice mutant line *cpm2* defective in the gene encoding allene oxide cyclase (*OsAOC*) (Biswas et al., 2003) was employed to investigate the possible association of JA signaling pathway with the growth stunting of blast-infected rice plants. The levels of endogenous JA and JA-isoleucine have been shown to be significantly reduced in *cpm2* compared with that in its WT plants (Riemann et al., 2013). Compared with WT line (Nihonmasari), *cpm2* plants exhibited more elongated leaves (**Figure 7A**). Blast infection resulted in reduction in leaf length of both *cpm2* and its WT plants (**Figure 7A**). However, *cpm2* had a remarkably higher relative leaf growth (leaf length in blast-infected plants relative to that in mock control plants) (**Figure 7B**), indicating that JA-reduction partially rescued the leaf growth inhibition caused by blast infection.

### Exogenous JA and ABA Caused Stunted Seedling Growth

The effect of each phytohormone on plant growth was examined by root drenching of rice seedlings with each phytohormone at four-leaf stage and measuring leaf lengths of the fifth leaf of the seedlings, 4 weeks after the onset of treatments. As shown in Supplementary Figure 1, GA treatment promoted elongated growth. In contrast, JA and ABA treatments severely suppressed plant growth. The inhibition effects of leaf growth by JA and ABA were fairly similar to those by blast infection (Supplementary Figure 1B). Other phytohormones showed only minor effects on leaf growth (Supplementary Figure 1).

## Discussion

As early as several decades ago, it was observed that rice plants infected with blast fungus *M. oryzae* exhibit a severe growth inhibition in addition to formation of blast disease lesions at the infection sites on leaf blades (Tokunaga et al., 1959). The growth inhibition was manifested the most in successive upper two to three leaves counted from the infected leaf (**Figure 1**), consistent with previous reports (Tokunaga et al., 1959; Yoshida et al., 1992). This morphological symptom (Zurikomi) may be considered a consequence of a trade-off between growth and defense, in which rice plants prioritized defense reactions at the expense of plant growth at the time of infection. However, the underlying mechanism for this phenomenon has remained essentially elusive. It was previously suggested that excess accumulation of coumarin (Tamari and Kaji, 1959a,b) or ethylene evolution (Kozaka and Teraoka, 1977) in response to blast infection might be responsible for the growth inhibition; however, these suggestions were not supported in later studies (Satoh and Kozaka, 1966, 1971; Yoshida et al., 1992). In this study, we found that cutting off the blast-infected leaf blade within 2 dpi significantly rescued the growth inhibition of upper distal leaves (**Figure 2**). This suggests that a yet unknown inhibitory substance(s) and/or signal is generated in the blast-infected sites and transmitted to upper uninfected distal leaves, where it induced growth inhibition. In the upper distal leaves, JA- and ABA-pathways were upregulated, whereas auxin- and GA-pathways were downregulated (**Figure 4**). It has been shown that JA and ABA negatively impact plant growth, in contrast to auxin and GAs, which promote plant growth (Swiatek et al., 2003; Xiang et al., 2008; Zhang and Turner, 2008; Yang et al., 2012; Liu et al., 2015; Hibara et al., 2016). The GA-responsive cell wall related genes (Budot et al., 2014) were also significantly downregulated (**Figure 6**). Further, the JA-deficient rice mutant *cpm2* showed a less pronounced leaf growth inhibition compared with its WT plants upon blast infection (**Figure 7**). In addition, treatment of rice seedlings with JA and ABA resulted in stunted growth resembling blast infection (Supplementary Figure 1). Taken together, these results strongly suggest that an inhibitory substance(s) and/or signal(s) generated in the blast-infected leaves activated JA and ABA, and suppressed GA and auxin signaling pathways in the upper distal leaves, which in turn resulted in growth stunting of the leaves. These findings provide new insights into the controlling mechanism of growth-defense balance in rice plants.

It has been reported that JA antagonistically interacts with the GA signaling cascade to prioritize defense over growth upon pathogen attacks in both *Arabidopsis* and rice (Yang et al., 2012). It was shown that *Arabidopsis* and rice *coi1* mutants, defective in JA perception, exhibited GA hypersensitivity; JA delayed GA-mediated degradation of DELLA protein, and conversely the *DELLA* mutant was less sensitive to JA in terms of growth inhibition (Yang et al., 2012). Our previous and present studies showed that the signaling pathways of both JA (**Figure 3**) and ABA (Jiang et al., 2010) are activated in blast-infected leaves. However, they differed in induction time: JA within 2 dpi, whereas ABA at 3 dpi. Taking these results together with the data shown in **Figures 2, 4–7**, it is tempting to speculate that JA may possibly take a role in signal transmission from blast-infected sites to upper distal leaves. Naturally, this hypothesis needs to be validated in future studies. Moreover, only a partial growth restoration in blast-infected *cpm2* seedlings (**Figure 7**) indicates the existence of additional factor(s) functioning in the growth stunting. In this context, ABA may also participate in the growth inhibition in later stages following the JA. Meanwhile, it is also possible that the partial growth restoration in *cpm2* seedlings is due to remaining quantities of JA (Riemann et al., 2013). The mechanism of distal activation of JA- and ABA-signaling pathways in response to blast infection is unknown. Determination of JA and ABA contents showed no appreciable changes either in blast-infected rice leaves (Schweizer et al., 1997; Jiang et al., 2013) or in upper distal leaves (Supplementary Table 2), even though the marker genes were activated in both the local (Schweizer et al., 1997; Jiang et al., 2010) and distal sites (**Figure 4**). A similar phenomenon was also observed for SA, in which blast infection of rice seedlings induced SA responsive genes without a concomitant increase in endogenous SA levels (Silverman et al., 1995). A possible explanation for these observations may be the intracellular relocalization or releasing from sequestration of these phytohormones in response to blast fungus infection. Alternatively, the possibility that a local increase of the phytohormones at the infection spots was technically difficult to detect cannot be excluded.

Stunted or dwarfed plant growth is also observed in rice plants infected with several disease-causing viruses, which has been associated with suppression of GA and/or auxin signaling cascades and expression of genes related to cell wall synthesis and expansion (Satoh et al., 2010, 2011, 2013; Budot et al., 2014). It has also been shown that the defense master regulator OsNPR1 attenuates rice plant growth by repressing auxin signaling pathway (Li et al., 2016). Consistent with these, we found significant reduction in auxin and GA pathways (**Figures 4E–H**) as well as in expression levels of cell wall related genes (**Figure 6**) in the upper distal leaves of blast-infected rice seedlings. This indicates that the reduction in auxin and GA pathways also plays a causative role in the growth stunting. Whether the reduction in auxin and GA pathways is associated with the upregulation of JA and ABA pathways remains to be elucidated.

Unexpectedly, SA responsive marker genes *WRKY45* and *OsNPR1*, and PR genes *OsPR1b* and *PBZ1* are downregulated in response to blast infection in the upper distal leaves (**Figure 5**). This may imply that rice plants prioritize the defense against infecting pathogen over the distal uninfected tissues as well in order to efficiently cope with the life-threatening situation. There have been several reports that priming of rice seedlings by pre-inoculation with an avirulent *M. oryzae* isolate enhanced resistance following infection by virulent isolates (Manandhar et al., 1998; Ashizawa et al., 2005; Yasuda et al., 2008; Filippi et al., 2014). However, to our knowledge, no study has yet explored the systemic defense regulation during rice–pathogen (*M. oryzae*) interaction. The downregulation of the *WRKY45* and *OsNPR1* may be due to the activation of ABA, as ABA negatively impacts on SA signaling pathway (Jiang et al., 2010; Sugano et al., 2010). It would be interesting to investigate how blast resistance changes in relation to alterations of phytohormone pathways in distal tissues upon blast infection.

In summary, we showed that a yet unknown inhibitory substance(s) and/or signal(s) is generated in the *M. oryzae*-infected leaves and transmitted to the upper distal leaves, where it causes growth stunting through activation of JA and ABA, and suppression of GA, auxin, and SA signaling pathways, to mediate the prioritizing of defense responses against pathogen attack over growth in rice plants.

## Author Contributions

C-JJ and Z-WL conceived and designed the study, X-LL, X-QL, HZ, and Y-JY conducted the experiments, collected and analyzed the data, and C-JJ wrote the manuscript.

## Conflict of Interest Statement

The authors declare that the research was conducted in the absence of any commercial or financial relationships that could be construed as a potential conflict of interest.
