# Supplementary material for: Stunted Growth Caused by Blast Disease in Rice Seedlings Is Associated with Changes in Phytohormone Signaling Pathways
Source: Front Plant Sci. 2017 Sep 6;8:1558. doi: 10.3389/fpls.2017.01558 (PMC5592330; doi:10.3389/fpls.2017.01558)
Supplement: Supplementary file 1 [file Table_1.pdf]

Supplementary Material

Stunted growth caused by blast disease in rice seedlings is associated with disturbed phytohormone balance

Chang-Jie Jiang[1,] *, Xiao-Long Liu[2], Hui Zhang[2], Ying-Jie Yu[2] and Zheng-Wei Liang[2,] *

*** Correspondence:** Corresponding Author: cjjiang@affrc.go.jp; liangzw@neigae.ac.cn

Supplementary Tables

Supplementary Table 1: Genes and primer sequences used for qRT-PCR analysis.

Gene name (RAP-DB ID)	Primer sequences (5'→3')	References
Rubq1 (Os06g0681400)	GGAGCTGCTGCTGTTCTAGG<br>TTCAGACACCATCAAACCAGA	Plant Sci. (2000) 28: 201-11.<br>doi: 10.1016/S0168-9452(00)00255-7
JAmyb (Os11g0684000)	TAGGGGTTCAAAGAGGACCA<br>TCCTCAGTGCAATTCTGGAG	MPMI (2001) 14: 527-35.<br>doi: 10.1094/MPMI.2001.14.4.527
OMT Os11g0303600)	TCGAGCGAGAGGAGCATGAA<br>ACCTAGCACTGGTGTGATTTTGTAG	Plant J. (2010) 61: 804-15.<br>doi: 10.1111/j.1365-313X.2009.04107.x
SalT (Os01t0348900)	CGAAATAATGTTCCATGGTGTT<br>TGTACTACGGATCGGTGCAA	Plant Physiol. (2003) 133: 1755-67.<br>doi: 10.1104/pp.103.025742
OsWsi18 (Os01g0705200)	TCACTTCAAATTCGGTGCAA<br>CACACCCGTCAGAAATCCTC	Plant Cell Physiol. (1998) 39: 64-72.<br>doi: 10.1093/oxfordjournals.pcp.a029290
OsARF1 (Os11g0523800)	GATGGTGCTTGTCCTTGGTG<br>CGGCTGACAACTTACCTCAC	Plant Mol. Biol. (2002) 50: 415-25.<br>doi: 10.1023/A:1019818110761
OsIAA9 (Os02g0805100)	GAAGGAGATGCATGCGTGAG<br>CATACATCAGTGCACACCCA	Func. Integr. Genomics (2006) 6: 47-59.<br>doi: 10.1007/s10142-005-0005-0
OsGA20ox1 (Os03g0856700)	TGGCGATTATTTCAGTTCCTAGC<br>CGGCCGGGATAGATTAGATAGA	Physiol. Plant (1997) 99: 111-8.<br>doi: 10.1111/j.1399-3054.1997.tb03438.x
OsGA2ox3 (Os01g0757200)	CCACGAGAAGATGAAGCTGC<br>AACCTGGCTGTACATTGTGC	J. Plant Res. (2003) 116: 161-4.<br>doi: 10.1007/s10265-003-0080-z
OsCESA5 (Os03g0837100)	ATCTGCCGTCTGGAATAGAG<br>ACCCACTCATCTCCAGTGTT	Front. Microbiol. (2014) 5: 1-9.<br>doi: 10.3389/fmicb.2014.00026
OsCESA6 (Os07g0252400)	CCATATATGCGGTGATACGA<br>CTTCCTTCCTTCTGTCCAAA	Front. Microbiol. (2014) 5: 1-9.<br>doi: 10.3389/fmicb.2014.00026
OsCSLA9 (Os06g0625700)	GTGTGCAAGTGCAAAGTTTC<br>GGTGAGCATATTGAGGAACC	Front. Microbiol. (2014) 5: 1-9.<br>doi: 10.3389/fmicb.2014.00026
OsEXP1 (Os04t0228400)	GTGTCCTTCTTCGTCTTCGT<br>ACTCCTCCATTACACCCAAA	Front. Microbiol. (2014) 5: 1-9.<br>doi: 10.3389/fmicb.2014.00026
OsEXP15 (Os03g0155700)	CGTCGTGGTAGTTGCAGTAG<br>TGCATTAATACTCCCGCATA	Front. Microbiol. (2014) 5: 1-9.<br>doi: 10.3389/fmicb.2014.00026
OsNPR1 (Os01t0194300)	TCCTCCTGGAGCTACTCACC<br>TCTGCGTTTCACAATTGACG	Plant Mol. Biol. (2010) 74: 549-62.<br>doi: 10.1007/s11103-010-9695-3
WRKY45 (Os05g0322900)	CGGGTAAACGATCGAAAGA<br>GCTGAGACGACACATCAACAA	Plant Cell (2007) 19: 2064-76.<br>doi: 10.1105/tpc.106.046250
OsPR1b (Os01g0382000)	ACGGGCGTACGTACTGGCTA<br>CTCGGTATGGACCGTGAAG	BBRC (2000) 278: 290-8.<br>doi: 10.1006/bbrc.2000.3781
PBZ1 (Os12g0555000)	GCGTTTGAGTCCGTGAGAGT<br>TCACCCATTGATGAAGCAAA	Biosci. Biotech. Biochem. (2001) 65: 205-8.<br>doi: 10.1271/bbb.65.205

Supplementary Table 2: Contents of JA and ABA in the 6th leaf of rice seedlings after inoculation with blast fungus *M. oryzae* (race 007.0)[a]

	Con	Blast	Blast-c
JA	131 ± 27	182 ± 66	155 ± 73
ABA	37 ± 2	40 ± 21	32 ± 6

[a] Rice seedlings at four-leaf stage were blast-inoculated (Blast), and half of them was subjected to cutting of the inoculated 4th leaf blades at 2-dpi (Blast-c). Whole 6th leaf from the seedlings was collected at 3-dpi. Con: mock control. Values are represented as mean ± standard deviation in picomoles per gram of fresh weight (n = 4 biological replicates).

Supplementary Figures

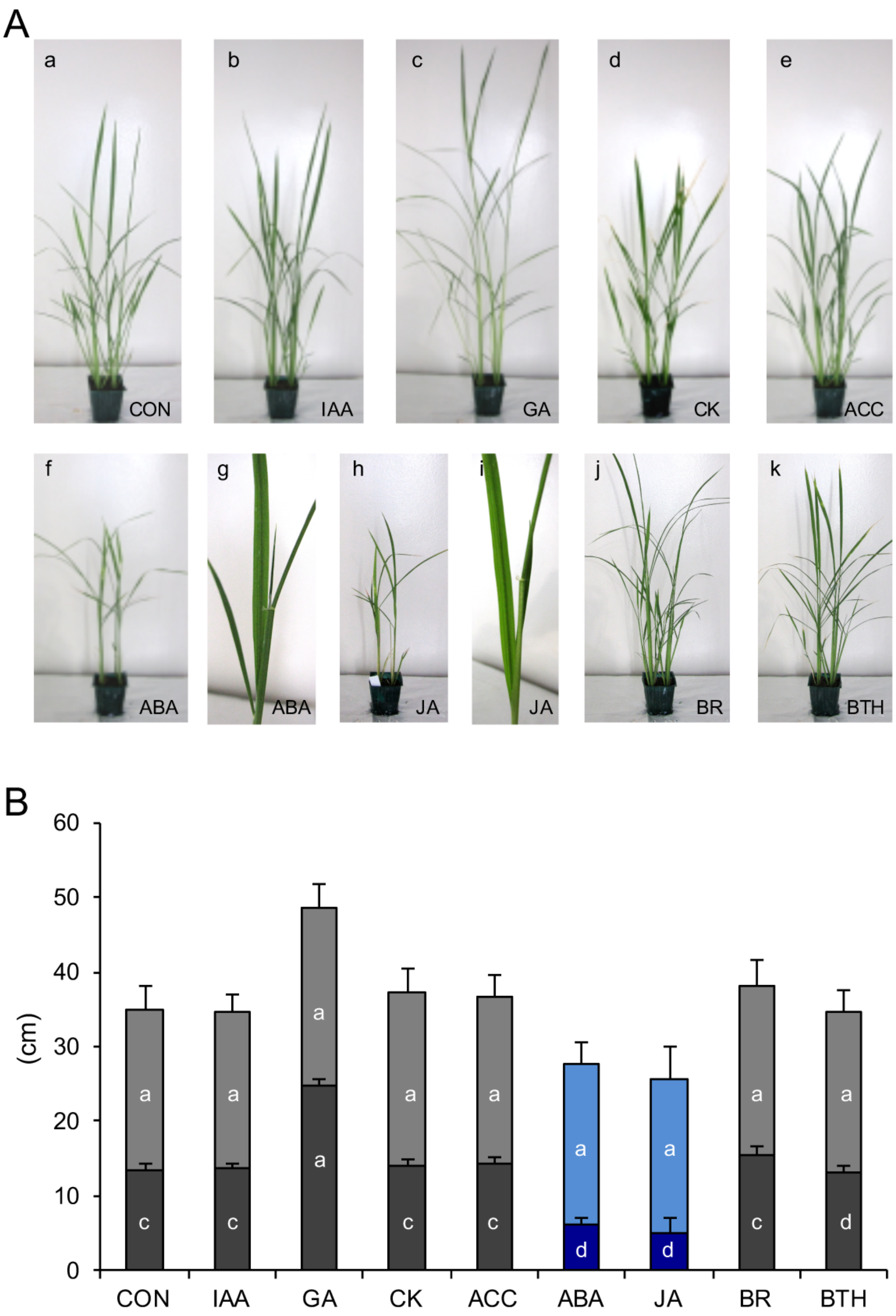

Supplementary Figure 1. Effect of exogenous phytohormones on rice seedling growth. Rice seedlings at four-leaf stage were treated with each indicated phytohormone by root drenching, and leaf lengths of the 5th leaf from each treatment were measured 4 weeks after the onset of treatments. (A) Image of rice seedlings treated with exogenous phytohormones. (B) Lengths of leaf blades (upper columns) and leaf sheaths (bottom columns) of the 5th leaf. Values are the means ± standard errors; different letters above bars indicate a significant difference ($P < 0.05$) based on Duncan's test.
